# Identification of small molecule drugs and development of a novel autophagy‐related prognostic signature for kidney renal clear cell carcinoma

**DOI:** 10.1002/cam4.3367

**Published:** 2020-08-11

**Authors:** Qianwei Xing, Chengjian Ji, Bingye Zhu, Rong Cong, Yi Wang

**Affiliations:** ^1^ Department of Urology Affiliated Hospital of Nantong University Nantong China; ^2^ Department of Urology The First Affiliated Hospital of Nanjing Medical University Nanjing China

**Keywords:** autophagy‐related genes, kidney renal clear cell carcinoma, prognostic index

## Abstract

Abnormal autophagic levels have been implicated in the pathogenesis of multiple cancers, however, its role in tumors is complex and has not yet been explored clearly. Hence, we aimed to explore the prognostic values of autophagy‐related genes (ARGs) for kidney renal clear cell carcinoma (KIRC). Differentially expressed ARGs and transcription factors (TFs) were identified in KIRC patients obtaining from the The Cancer Genome Atlas (TCGA) database. Then, networks between TFs and ARGs, gene ontology functional annotations and Kyoto Encyclopedia of Genes and Genomes pathway enrichment analysis were conducted. Next, we performed consensus clustering, COX regression analysis and Lasso regression analysis to identify the prognostic ARGs. Finally, an individual prognostic index (PI, *riskScore*) was established. Based on TCGA cohort and ArrayExpress cohort, Survival analysis, ROC curve, independent prognostic analysis, and clinical correlation analysis were also performed to evaluate this PI. Based on differentially expressed ARGs, KIRC patients were successfully divided into two clusters (*P* = 5.916e‐04). AS for PI, it was constructed based on 11 ARGs and significantly classified KIRC patients into high‐risk group and low‐risk group in terms of OS (*P* = 4.885e‐15 for TCGA cohort, *P* = 6.366e‐03 for ArrayExpress cohort). AUC of its ROC curve reached 0.747 for TCGA cohort and 0.779 for ArrayExpress cohort. What's more, this PI was proven to be a valuable independent prognostic factor in both univariate and multivariate COX regression analysis (*P* < .001). Prognostic nomograms were also performed to visualize the relationship between individual predictors and survival rates in patients with KIRC. By means of connectivity map database, emetine, cephaeline and co‐dergocrine mesilate related to ARGs were found to be negatively correlated with KIRC. This study provided an effective PI for KIRC and also displayed networks between TFs and ARGs. KIRC patients were successfully divided into two clusters based on differentially expressed ARGs. Besides, small molecule drugs related to ARGs were also identified for KIRC.

## Graphical abstract

The strength of this article was that we performed a systematic analysis of the roles of autophagy in kidney renal clear cell carcinoma (KIRC) with a robust statistical approach. A novel prognostic index (PI) was successfully established and carefully evaluated. Moreover, networks between autophagy‐related genes (ARGs) and transcription factors were constructed for future basic research. Tumor clustering based on ARGs was effective, indicating ARGs played a vital role in KIRC. Last but not least, small molecule drugs related to ARGs were also identified for KIRC.

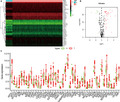

## 1 INTRODUCTION

Autophagy as a conservative metabolic way to maintain the homeostasis of the cell environment in the body, it can be expressed in all cells and selectively recover necrotic, injured and genetically deficient cells or tissues, providing energy for the body or regulating the stability of organ function.^1^ However, the definite role of autophagy in tumors is complex and has not yet been explored clearly. Autophagy has different mechanisms in different types of cancer, tumor microenvironments, tumor stages and so on.^2^, ^3^ Jan Karlseder of the Salk Institute in the United States in a recent study further explained the mechanism by which autophagy inhibits cancer in the early stages of cancer that it was closely related to the ‘replication crisis’ caused by telomere fusion during cell division.^4^ With the development of cancer, the role of autophagy in tumor would change from inhibiting cancer to promote cancer survival.^5^, ^6^ Even in established tumors, autophagy could also help tumor cells resist a variety of drug treatments.^7^, ^8^

Globally, there were 404,300 new cases of renal cell cancer and 175,100 new deaths of this tumor in 2018, ranking 16th in morbidity and mortality among all cancers, making it the third most common tumor in the urinary system.^9^ Although most renal cell carcinoma (RCC) developed slowly, many patients would already have metastasis at the time of detection due to the lack of typical symptoms.^10^ Moreover, renal cancer cells lacked sensitivity to radiotherapy and chemotherapy. Surgery remained the mainstay of therapy, however there were still 1/4 of these patients having a recurrence or metastasis after surgical treatment.^11^, ^12^ Due to the lack of accurate predictive markers for the prognosis of patients with RCC, the establishment of an effective prognosis prediction model is of great significance for the management of patients in the whole course of the disease.

Thanks to the availability of high‐throughput expression data nowadays, it has become feasible for us to use public database data for analyzing the associations between autophagy‐related genes (ARGs) and the clinical outcomes of kidney renal clear cell carcinoma (KIRC) patients. Here, we explored the associations between ARGs and transcription factors (TFs) and constructed prognosis prediction indexes for KIRC, based on related transcriptome profiling data and clinical information downloaded from the The Cancer Genome Atlas (TCGA) database. Our study was anticipated to provide new insights of autophagy for future work.

## 2 MATERIALS AND METHODS

### 2.1 Acquisition and preparation of data

Transcriptome profiling data and related clinical information of KIRC were downloaded from TCGA Data Portal (https://tcga-data.nci.nih.gov/tcga/; accessed August 2019) and ArrayExpress (https://www.ebi.ac.uk/arrayexpress/; accessed March 2020). The Human Autophagy Database (HADb, http://autophagy.lu/clustering/index.html) is a dedicated database reserving human ARGs. We did an overlap by comparing the obtained RNA‐seq data with the HADb database. Then, the RNA‐seq data were background corrected and standardized by the R programming language.

### 2.2 Identification and enrichment analysis of differently expressed ARGs

Differently expressed ARGs were carried out by using “Lima” package in R statistical software between KIRC and solid tissue normal samples. The threshold for identification of ARGs was set as adjusted *P*‐value (FDR) < .05 and |log2fold changes (FC)| > 1. Gene functional enrichment analyses, including gene ontology (GO) functional annotations and Kyoto Encyclopedia of Genes and Genomes (KEGG) pathway enrichment analysis, were conducted to analyze the biological functions, cellular localization, and signaling pathways of targeted genes. In this study, we performed GO and KEGG enrichment analysis on differentially expressed ARGs by using the “clusterProfiler” R package.

### 2.3 Identification of differently expressed TFs and construction of a network between TFs and ARGs

The Cistrome database (http://www.cistrome.org/) is a comprehensive resource for predicted TF targets and enhancer profiles in cancers. The prediction was from integrative analysis of TCGA expression profiles and public ChIP‐seq profiles. Differently expressed TFs were carried out by using “Lima” package in R statistical software between KIRC and solid tissue normal samples. Correlation test between differently expressed TFs and ARGs was performed by R programming language. Moreover, correlation coefficient at least 0.4 corresponding to a *P* < .01 were selected as the significantly correlated.

### 2.4 Cluster analysis

In order to show whether autophagy has an important impact on the overall prognosis of patients with KIRC, consensus clustering was performed to divide patients into clusters based on the differently expressed ARGs. “ConsensusClusterPlus” package in R statistical software was adopted to perform the Consensus clustering.

### 2.5 Establishment of an independent prognostic index (PI, *riskScore*) based on ARGs

In order to identify the key ARGs, a univariate COX regression analysis was firstly performed by us to exclude some ARGs with little prognostic value. Subsequently, a multivariate Cox regression analysis was utilized to remove the genes that might not be an independent indicator in prognosis monitoring. In addition, in order to prevent the occurrence of overfitting, we also used Lasso regression to remove key ARGs highly correlated with one other. According to the weight of each gene in Lasso regression analysis, we finally obtained the correlation coefficient in the model formula for predicting the prognosis of patients. Combined with the expression of various prognosis‐related genes, we established an independent prognostic model. The PI (*riskScore*) was calculated using the following formula β1 × gene1 expression + β2 × gene2 expression + ⋯ + βn × genen expression, where β corresponded to the correlation coefficient.

### 2.6 Evaluation of the prognostic index in TCGA cohort and ArrayExpress cohort

According to our prognostic model, each patient in TCGA cohort and ArrayExpress cohort will get a risk score. In each cohort, we set the median risk score as the cutoff value for dividing KIRC patients into a high‐risk group and a low‐risk group, respectively. Kaplan‐Meier (K‐M) method was utilized to plot the survival curves, and the log‐rank test was performed to assess differences in the survival rates between high‐risk group and low‐risk group. The receiver operating characteristic curves (ROC) were created by the “survivalROC” package, and the area under the curve (AUC) values was calculated to evaluate the specificity and sensitivity of the model. The *riskScore* distribution of patients in different risk groups, the number of censored patients, and the heatmap of prognosis‐related ARGs were also displayed. A prognostic nomogram was also performed to visualize the relationship between individual predictors and survival rates in patients with KIRC based on the Cox proportional hazard regression model by means of “rms” package of R software. C‐index and the Calibration curves were used to evaluated the performance of the prognostic nomogram.

To further evaluate whether our model can be used as an independent prognostic factor, we included age, gender, stage, race, grade, T, M, N, and PI as independent variables. And then we did univariate cox regression analysis and multivariate cox regression analysis on the changes of survival time and survival outcome. Multivariate ROC curves were also made to evaluate the prognostic value of each variable. Finally, we combined various clinical variables and *riskScore* to make a new nomogram to predict the survival outcome of patients in different cohorts.

In addition, we also made a clinical correlation analysis to analyze the correlation between PI and clinical features such as age, gender, stage, race, grade, T, M, N. Besides, the correlation between each prognosis‐related ARGs and clinical features such as age, gender, stage, race, grade, T, M, N were also analyzed.

### 2.7 Identification of candidate small molecule drugs

Connectivity map (cMap), as a gene expression profiles database led by Todd Golub and Eric Lander, it facilitated researchers to quickly identify molecule drugs highly correlated with diseases and discover its possible mechanism.^13^ Up‐regulated and down‐regulated ARGs related to KIRC were uploaded and then functional connection between genes and bioactive chemicals was explored. Connectivity scores ranging from −1 to 1 were utilized to estimate how closely a compound is connected to the query signature. Positive score indicated that the query signature could be promoted by a drug, while a negative score could be repressed by a drug in cMap.

### 2.8 Statistical analysis

Statistical analyses of all data utilized in this article were completed by R software (version 3.4.1, https://www.r-project.org/). When the difference met a joint satisfaction of FDR < 0.05 and |log2FC| > 1, it was regarded to be statistically significant. “ConsensusClusterPlus” package was adopted to perform the Consensus clustering. The univariate and multivariate COX regression analysis were used to evaluate the relationship between ARGs expression and survival data to establish a prognostic model. “rms” package of R software was used to create the nomogram. The receiver operating characteristic curves were created by the “survivalROC” package of R and AUC values were also calculated by this package too. All statistical tests were two‐sided and *P* < .05 was considered to be statistically significant.

## 3 RESULTS

### 3.1 Differentially expressed ARGs

**Figure 1:**
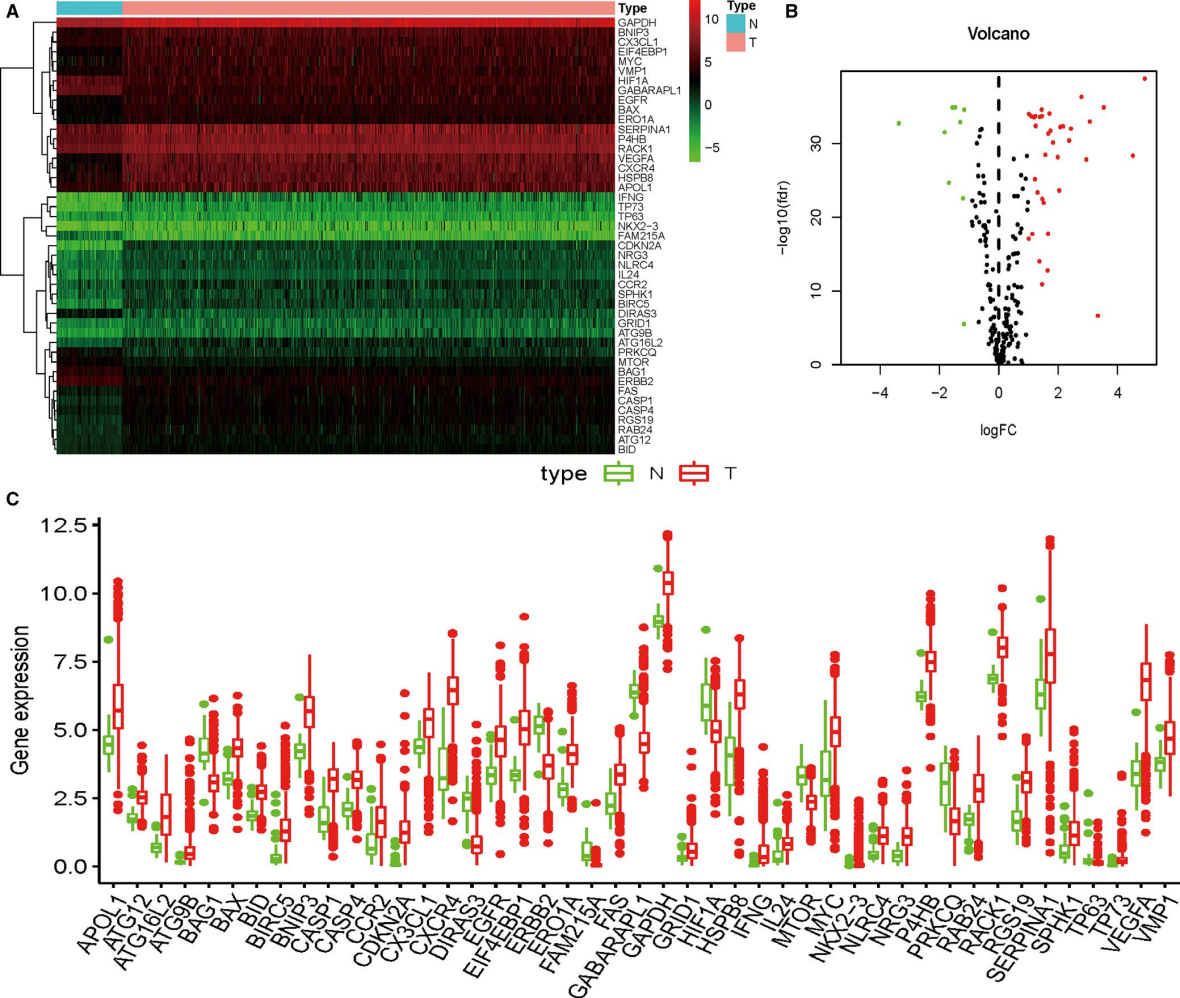
Differentially expressed autophagy‐related genes (ARGs); A, Heatmap of differentially expressed ARGs; B, Volcano map of differentially expressed ARGs; C, Boxplot of differentially expressed ARGs

The flow diagram for this study was displayed in Figure S1. Through the online TCGA database, we obtained the RNA sequences and clinical information of 539 KIRC samples and matched 72 solid tissue normal samples. By comparing autophagy‐related genes from HADb, we finally obtained the expression of 232 relevant genes. In order to further screen out valuable differentially expressed ARGs, we set the joint satisfaction of FDR < 0.05 and |log2FC| > 1 to the filtration condition. Heatmap of differently expressed ARGs was presented in Figure 1A. Figure 1B was a volcano map showing 9 down‐regulated and 36 up‐regulated differentially expressed ARGs. Boxplot of these ARGs was detailed in Figure 1C.

### 3.2 Functional annotation of differentially expressed ARGs

**Figure 2:**
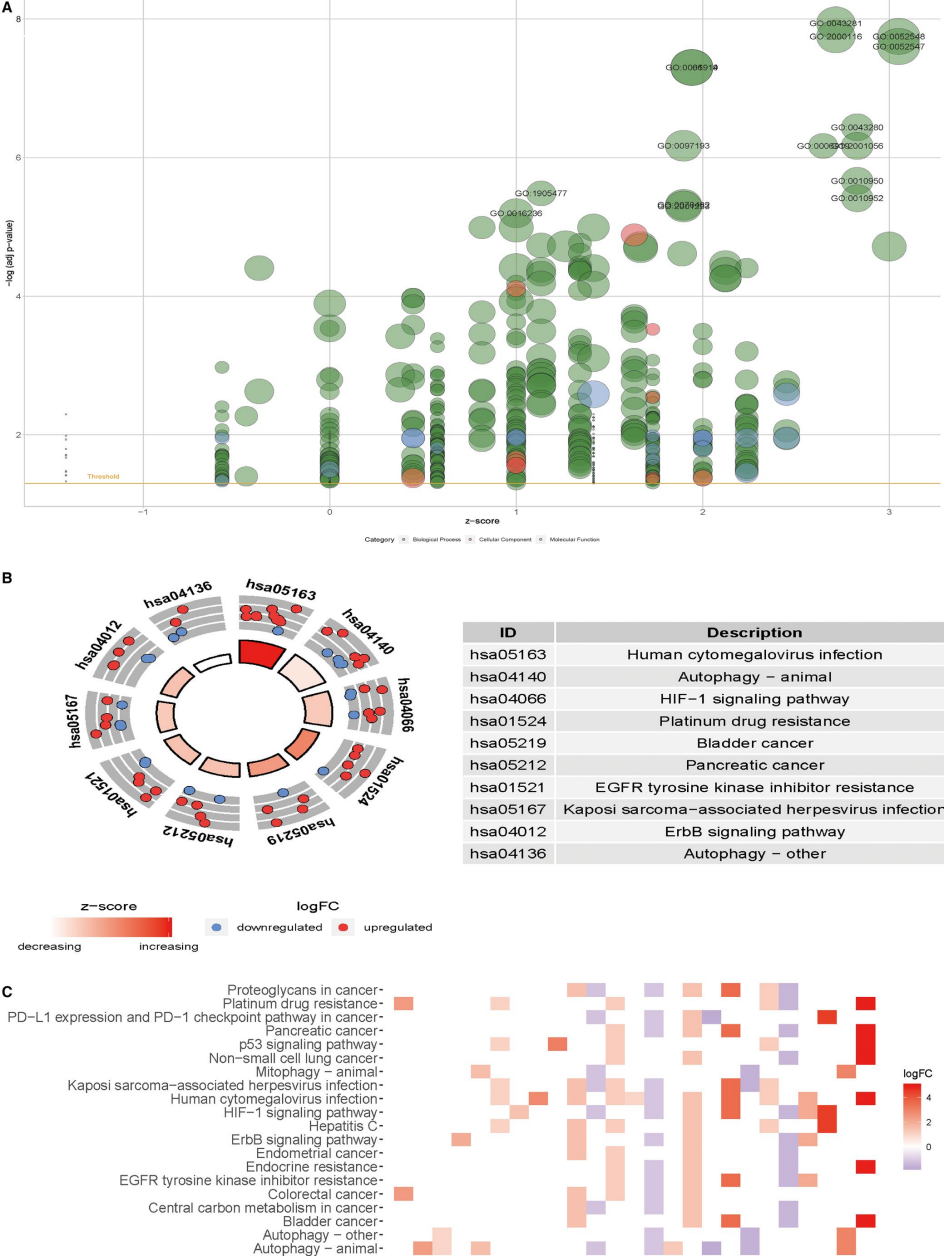
Functional annotation of differentially expressed autophagy‐related genes (ARGs); A, The bubble plot of enriched gene ontology (GO) terms. Greed circles correspond to the biological process, red indicates the cellular component, and blue shows the molecular function category. B, Circle diagram of Kyoto Encyclopedia of Genes and Genomes (KEGG) pathways. Red circles display up‐regulation and blue ones down‐regulation; C, Heatmap of KEGG pathways; The color of each block depends on the logFC values

In order to better understand the functions and mechanisms of these ARGs, we analyzed the enrichment of GO terms function and KEGG pathway. The results of the functional enrichment analysis are summarized in Figure 2. Table 1 lists the top 10 main GO entries and the KEGG pathways. In terms of biological processes, these differential genes are mainly concentrated in autophagy, regulation of peptidase, and endopeptidase activity and so on. Separately, autophagosome is the highest enrichment level in GO terms for cellular components, protein heterodimerization activity, and peptidase regulator activity were most enriched GO terms for molecular function (Figure 2A; Table 1). In addition, the results of KEGG pathway enrichment analysis were shown in Figure 2B, which shows that these differentially expressed ARGs were closely related to Human cytomegalovirus infection, Autophagy animal, HIF‐1 signaling pathway, and other functional pathways. We also show the correlation between these differentially expressed ARGs and the related pathways in the form of heatmap (Figure 2C).

**Table 1:** Gene ontology (GO) and Kyoto Encyclopedia of Genes and Genomes (KEGG) analysis of differentially expressed autophagy‐related genes (ARGs)

Category	ID	Term	*P*‐value	Genes
BiologicalProcess	GO:0043 281	Regulation of cysteine‐type endopeptidase activity involved in apoptotic process	.0000000	BAX/FAS/CASP4/NLRC4/MYC/VEGFA/TP63/CASP1/BID/RACK1/BIRC5
BiologicalProcess	GO:0052 548	Regulation of endopeptidase activity	.0000000	BAX/FAS/CASP4/NLRC4/MYC/VEGFA/TP63/CASP1/BID/GAPDH/RACK1/BIRC5/SERPINA1
BiologicalProcess	GO:2000 116	Regulation of cysteine‐type endopeptidase activity	.0000000	BAX/FAS/CASP4/NLRC4/MYC/VEGFA/TP63/CASP1/BID/RACK1/BIRC5
BiologicalProcess	GO:0052 547	Regulation of peptidase activity	.0000000	BAX/FAS/CASP4/NLRC4/MYC/VEGFA/TP63/CASP1/BID/GAPDH/RACK1/BIRC5/SERPINA1
BiologicalProcess	GO:0006 914	Autophagy	.0000000	RAB24/IFNG/ATG12/BNIP3/CASP1/RGS19/HIF1A/VMP1/GAPDH/ATG9B/ATG16L2/MTOR/GABARAPL1
BiologicalProcess	GO:0061 919	Process utilizing autophagic mechanism	.0000000	RAB24/IFNG/ATG12/BNIP3/CASP1/RGS19/HIF1A/VMP1/GAPDH/ATG9B/ATG16L2/MTOR/GABARAPL1
BiologicalProcess	GO:0097 193	Intrinsic apoptotic signaling pathway	.0000000	BAX/CASP4/BNIP3/TP73/P4HB/ERO1A/TP63/HIF1A/BID/RACK1
BiologicalProcess	GO:0070 482	Response to oxygen levels	.0000000	FAS/MYC/BNIP3/P4HB/VEGFA/ERO1A/CXCR4/CASP1/HIF1A/MTOR
BiologicalProcess	GO:2001 233	Regulation of apoptotic signaling pathway	.0000000	BAX/FAS/BNIP3/TP73/P4HB/TP63/HIF1A/BID/CX3CL1/RACK1
BiologicalProcess	GO:1904 951	Positive regulation of establishment of protein localization	.0000002	IFNG/TP73/ERBB2/TP63/CASP1/HIF1A/BID/GAPDH/EGFR/RACK1
CellularComponent	GO:0005 776	Autophagosome	.0000001	RAB24/ATG12/VMP1/ATG9B/ATG16L2/GABARAPL1
CellularComponent	GO:0000 421	Autophagosomemembrane	.0000010	VMP1/ATG9B/ATG16L2/GABARAPL1
CellularComponent	GO:0061 702	Inflammasomecomplex	.0000058	CASP4/NLRC4/CASP1
CellularComponent	GO:0000 407	Phagophore assembly site	.0000740	ATG12/VMP1/ATG9B
CellularComponent	GO:0005 741	Mitochondrialouter membrane	.0008724	BAX/BNIP3/BID/MTOR
CellularComponent	GO:0031 968	Organelle outer membrane	.0013680	BAX/BNIP3/BID/MTOR
CellularComponent	GO:0019 867	Outermembrane	.0014187	BAX/BNIP3/BID/MTOR
CellularComponent	GO:0005 774	Vacuolarmembrane	.0030639	VMP1/ATG9B/ATG16L2/MTOR/GABARAPL1
CellularComponent	GO:0005 793	Endoplasmicreticulum‐Golgiintermediatecompartment	.0032226	P4HB/VMP1/SERPINA1
CellularComponent	GO:0044 445	Cytosolicpart	.0032461	CASP4/NLRC4/CASP1/RACK1
MolecularFunction	GO:0046 982	Proteinheterodimerizationactivity	.0000248	BAX/BNIP3/P4HB/VEGFA/ERBB2/HIF1A/BID/EGFR
MolecularFunction	GO:0061 134	Peptidase regulatoractivity	.0000143	NLRC4/CASP1/GAPDH/RACK1/BIRC5/SERPINA1
MolecularFunction	GO:0004 857	Enzyme inhibitor activity	.0003877	NLRC4/CDKN2A/GAPDH/RACK1/BIRC5/SERPINA1
MolecularFunction	GO:0005 126	Cytokine receptor binding	.0005663	IFNG/VEGFA/BID/CX3CL1/CCR2
MolecularFunction	GO0031 625	Ubiquitin protein ligase binding	.0009118	CXCR4/HIF1A/BID/EGFR/GABARAPL1
MolecularFunction	GO0044 389	Ubiquitin‐like protein ligase binding	.0011111	CXCR4/HIF1A/BID/EGFR/GABARAPL1
MolecularFunction	GO0048 018	Receptor ligand activity	.0057178	IFNG/IL24/VEGFA/CX3CL1/NRG3
MolecularFunction	GO0019 903	Protein phosphatase binding	.0002023	SPHK1/ERBB2/EGFR/RACK1
MolecularFunction	GO0019 902	Phosphatasebinding	.0007477	SPHK1/ERBB2/EGFR/RACK1
MolecularFunction	GO0004 866	Endopeptidaseinhibitoractivity	.0008342	NLRC4/GAPDH/BIRC5/SERPINA1
KEGG PATHWAY	hsa05163	Humancytomegalovirusinfection	.0000000	BAX/FAS/CDKN2A/MYC/VEGFA/CXCR4/BID/EIF4EBP1/CX3CL1/EGFR/MTOR
KEGG PATHWAY	hsa04140	Autophagy ‐ animal	.0000000	ATG12/BNIP3/HIF1A/VMP1/ATG9B/PRKCQ/ATG16L2/MTOR/GABARAPL1
KEGG PATHWAY	hsa04066	HIF‐1 signaling pathway	.0000000	IFNG/VEGFA/ERBB2/HIF1A/EIF4EBP1/GAPDH/EGFR/MTOR
KEGG PATHWAY	hsa01524	Platinum drug resistance	.0000013	BAX/FAS/CDKN2A/ERBB2/BID/BIRC5
KEGG PATHWAY	hsa05219	Bladdercancer	.0000015	CDKN2A/MYC/VEGFA/ERBB2/EGFR
KEGG PATHWAY	hsa05212	Pancreaticcancer	.0000015	BAX/CDKN2A/VEGFA/ERBB2/EGFR/MTOR
KEGG PATHWAY	hsa01521	EGFR tyrosine kinase inhibitor resistance	.0000021	BAX/VEGFA/ERBB2/EIF4EBP1/EGFR/MTOR
KEGG PATHWAY	hsa05167	Kaposisarcoma‐associatedherpesvirusinfection	.0000026	BAX/FAS/MYC/VEGFA/HIF1A/BID/MTOR/GABARAPL1
KEGG PATHWAY	hsa04012	ErbB signaling pathway	.0000032	MYC/ERBB2/EIF4EBP1/EGFR/MTOR/NRG3
KEGG PATHWAY	hsa04136	Autophagy ‐ other	.0000164	ATG12/ATG9B/MTOR/GABARAPL1

### 3.3 Differentially expressed TFs

**Figure 3:**
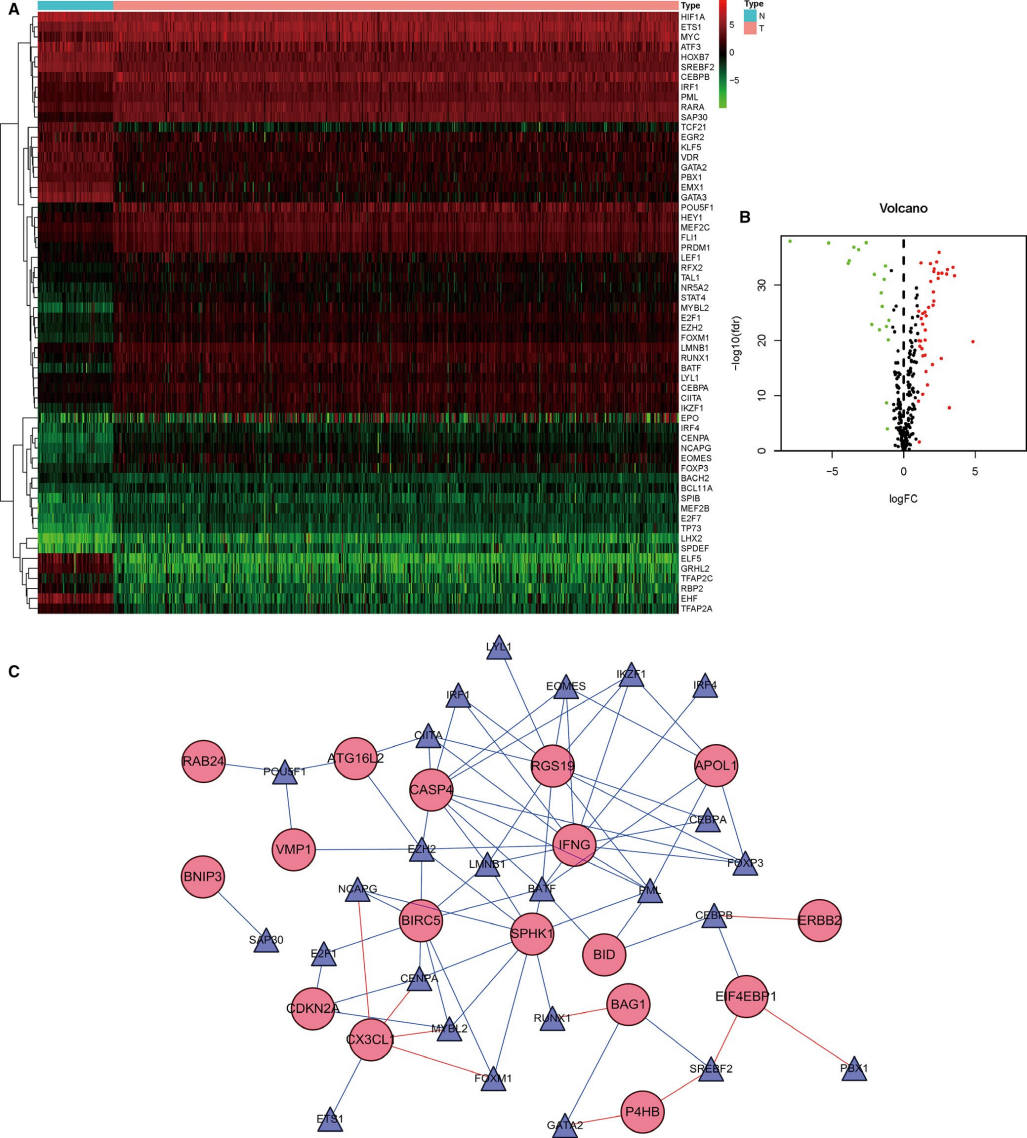
Differentially expressed transcription factors (TFs); A, Heatmap of differentially expressed TFs; B, Volcano map of differentially expressed TFs; C, A network shows the relationship between TFs and ARGs

By comparing genetic sequences data with TFs from Cistrome, we finally obtained the expression of 317 relevant TFs. In order to further screen out valuable differentially expressed TFs, we set the joint satisfaction of FDR < 0.05 and |log2FC| > 1 to the filtration condition. Heatmap of differently expressed ARGs was presented in Figure 3A. Figure 3B was a volcano map showing 19 down‐regulated and 41 up‐regulated differentially expressed TFs. Networks between TFs and ARGs were detailed in Figure 3C.

### 3.4 Identification of clusters for KIRC based on ARGs

**Figure 4:**
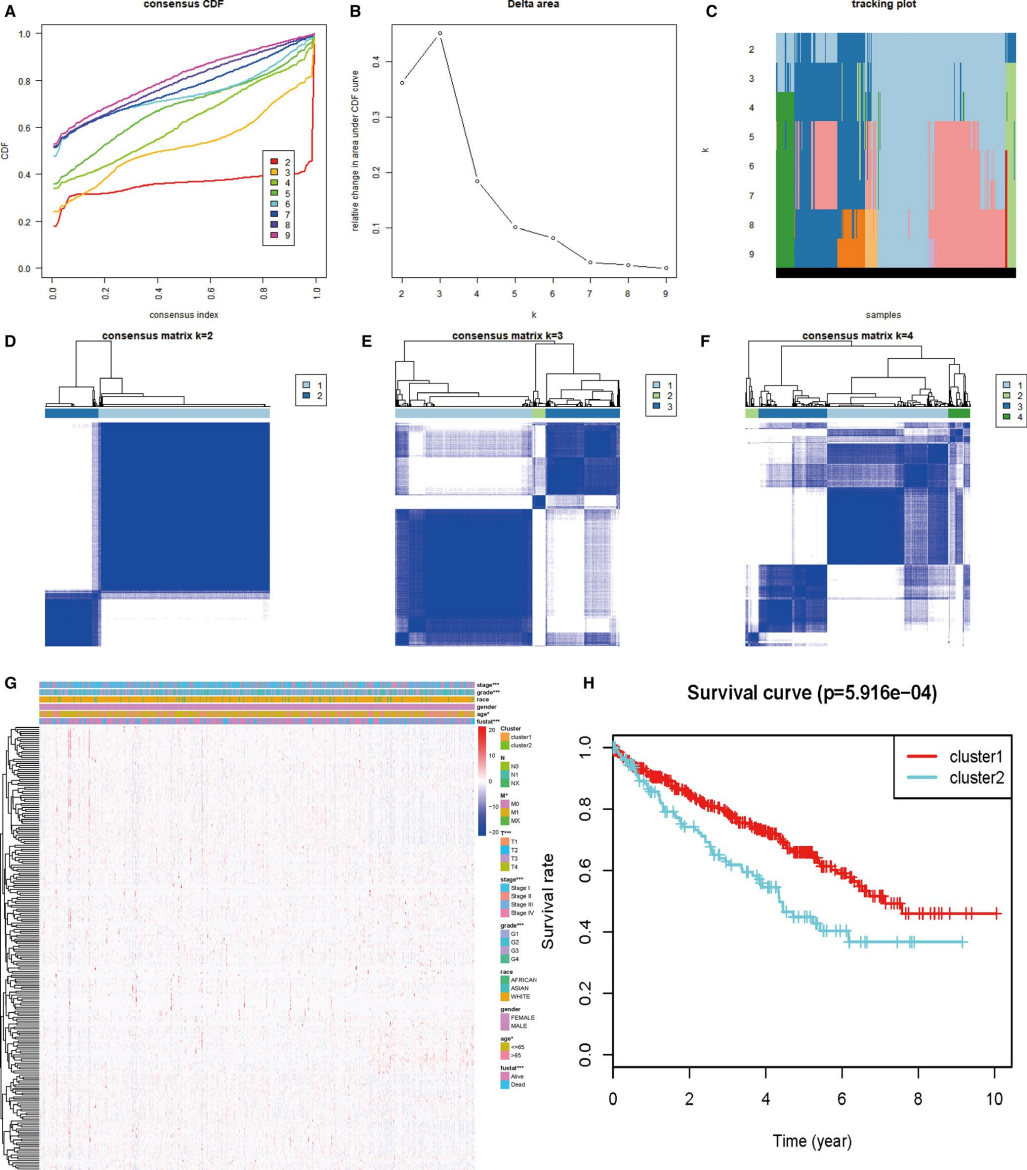
Identification of two clusters of kidney renal clear cell carcinoma (KIRC) patients that exhibited distinct ARG features and clinical outcomes using consensus clustering; A, Cumulative distribution function for *k* = 2 to 9; B, Relative change in the area under the CDF curve for *k* = 2 to 9. C, Tracking plot for *k* = 2 to 9. D–F, Consensus clustering matrix for *k* = 2, 3, and 4. G, Heatmap of the consensus matrix. *P < .05; ***P < .001; H, Kaplan‐Meier OS curves for the KIRC patients stratified by two clusters

Furthermore, we did consensus clustering for patients with KIRC based on ARGs. Figure 4A‐C suggested that satisfactory clustering effect could be obtained when *k* = 3. However, Figure 4D‐E suggested that *k* = 2 is the best option. Finally, patients with KIRC were divided into two groups (cluster 1 and cluster 2). Then, the clinical characteristics and survival curves of these two groups were analyzed. From Figure 4G, we found that there was a significant correlation between tumor stage, grade, age, fustat, and clustering. According to Kaplan‐Meier analysis, we noticed that the survival of patients in cluster 2 was worse than that in cluster 1 (Figure 4H). Considering the distribution of clinical features and survival curve, this clustering method had a certain significance.

### 3.5 Construction of a prognostic model index (PI, *riskScore*) based on ARGs

Based on the obtained differentially expressed ARGs, we carried out univariate and multivariate COX regression analysis, respectively, to evaluate the prognostic value of these ARGs (Figure S2A,B). According to the results of multivariate cox regression analysis, we obtained 11 risk ARGs. To avoid overfitting the model, we further took Lasso regression (Figure 2C‐D). Finally, 11 risk ARGs were obtained. According to the coefficient of each differentially expressed ARGs in Lasso regression, we then constructed a PI to predict the prognosis of patients with KIRC. The 11 prognostic ARGs related PI formula was as follows: *riskScore* = CASP4 expression × 0.409410245939865 + IFNG expression × 0.247091026343113 + BAG1 expression × (−0.31339800616801) + BNIP3 expression × (−0.312754657270375) + ERBB2 expression × 0.230285057472967 + RGS19 expression × (−0.336769784907294) + BID expression × 0.553711988544078 + EIF4EBP1 expression × 0.23902996965133 + CX3CL1 expression × (−0.26126419480746) + PRKCQ expression × (−0.409509859853768) + ATG16L2 expression × 0.241519437514572.

**Figure 5:**
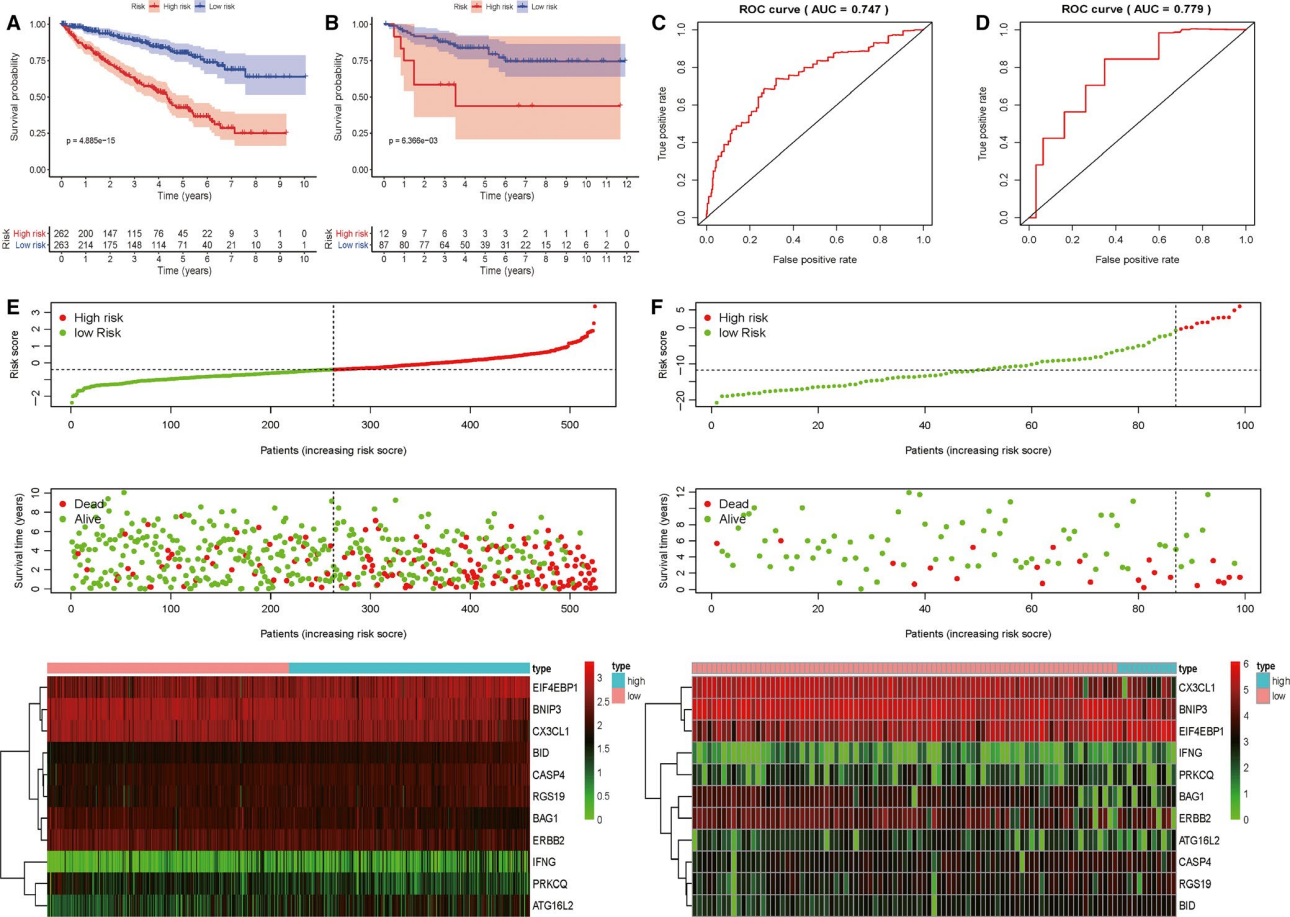
Evaluation of prognostic index (*riskScore*) based on autophagy‐related genes (ARGs) for kidney renal clear cell carcinoma (KIRC) patients; A, Kaplan‐Meier plot based on TCGA cohort; B, Kaplan‐Meier plot based on ArrayExpress cohort; C, ROC curve based on TCGA cohort; D, ROC curve based on ArrayExpress cohort; E, Clinical characteristics in TCGA database (in order from top to bottom): The risk score distribution of KIRC patients in high and low risk groups; The overall survival status distribution of KIRC patients with increasing risk score; The heatmap of the 11 key genes expression profiles in the TCGA dataset; F, Clinical characteristics in ArrayExpress database (in order from top to bottom): The risk score distribution of KIRC patients in high and low risk groups; The overall survival status distribution of KIRC patients with increasing risk score; The heatmap of the 11 key genes expression profiles in the ArrayExpress dataset

**Figure 6:**
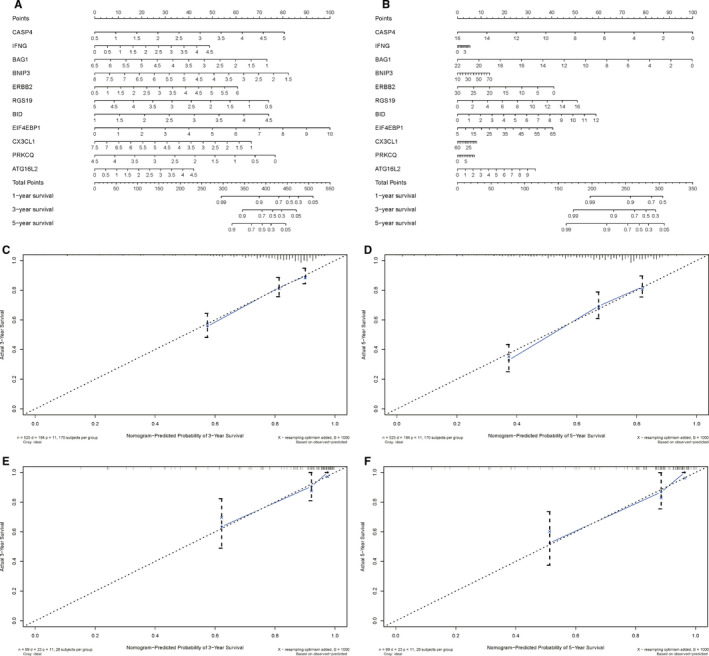
Diagnostic nomograms to clarify the relationship between risk genes and overall survival; A, A nomogram for TCGA cohort; B, A nomogram for ArrayExpress cohort; C, the Calibration curve of nomogram‐predicted probability of 3‐Year survival based on TCGA cohort; D, the Calibration curve of nomogram‐predicted probability of 5‐year survival based on TCGA cohort; E, the Calibration curve of nomogram‐predicted probability of 3‐year survival based on ArrayExpress cohort; F, the Calibration curve of nomogram‐predicted probability of 5‐year survival based on ArrayExpress cohort

After obtaining the PI (*riskScore*) based on ARGs for predicting the prognosis of KIRC patients, we got the *riskScore* of each patient in TCGA cohort. Then, we divided patients into two groups (high‐risk group and low‐risk group) according the median *riskScore*. Next, we evaluated this model in TCGA cohort from the following aspects: clinical characteristics, survival curve, ROC curve, and prognostic nomogram (Figures 5 and 6). Figure 5E showed that the higher the risk scores, the higher the patients in high‐risk group, and the higher the numbers of dead persons. The heatmap of these 11 key genes expression profiles in the TCGA dataset was also detailed in this figure. Kaplan‐Meier plot represents that patients in the high‐risk group had significantly shorter overall survival time than those in the low‐risk group (*P* = 4.885e‐15, Figure 5A). From the ROC curve of Figure 5C, AUC of this model for predicting prognosis reached 0.747, having a moderate prediction accuracy. In addition, a prognostic nomogram was created to quantify the relationship between these risk genes and survival. From this nomogram, we could obtain the total points and estimate the 1‐year, 2‐year, and 3‐year survival rate of each patient (Figure 6A). Table 2 showed the evaluation results for this nomogram (the C‐index and the AUC). The Calibration curves (Figure 6C‐D) further clarified the accuracy of this nomogram.

**Table 2:** Evaluation results of nomograms

Cohort	Nomogram composed of risk genes				Nomogram composed of clinical characteristics and *riskScore*			
	C‐index	AUC of 1‐y ROC	AUC of 3‐y ROC	AUC of 5‐y ROC	C‐index	AUC of 1‐y ROC	AUC of 3‐y ROC	AUC of 5‐y ROC
TCGA cohort	0.7149080	0.744	0.729	0.760	0.8033716	0.861	0.806	0.800
ArrayExpress cohort	0.8278069	0.800	0.850	0.834	0.8726003	0.895	0.897	0.861

### 3.6 Verification of the model in external cohort

In order to verify whether our model was reliable, we used it to analyze the external cohort from ArrayExpress database (E‐MTAB‐1980). The external cohort contained 101 KIRC patients. Similarly, we calculated the *riskScore* of each patient based on PI, and divided the patients into high‐risk group and low‐risk group according to the cut‐off value we obtained in the TCGA cohort. A Kaplan‐Meier curve based on the log‐rank test and the ROC curve were created to visualize the prognostic value of our established prognostic model in external cohort (Figure 5B,D). The areas under the ROC (AUC) values of *PI* was 0.779. Figure 5F showed that the higher the risk scores, the higher the patients in high‐risk group, and the higher the numbers of dead persons. The heatmap of these 11 key genes expression profiles in the external cohort was also detailed in this figure. In addition, a prognostic nomogram was also created to quantify the relationship between these risk genes and survival in external cohort (Figure 6B). The corresponding evaluation results of this nomogram are shown in Table 2 and Figure 6E, F.

### 3.7 Independent prognostic factor evaluation and correlation with clinical characteristics

**Figure 7:**
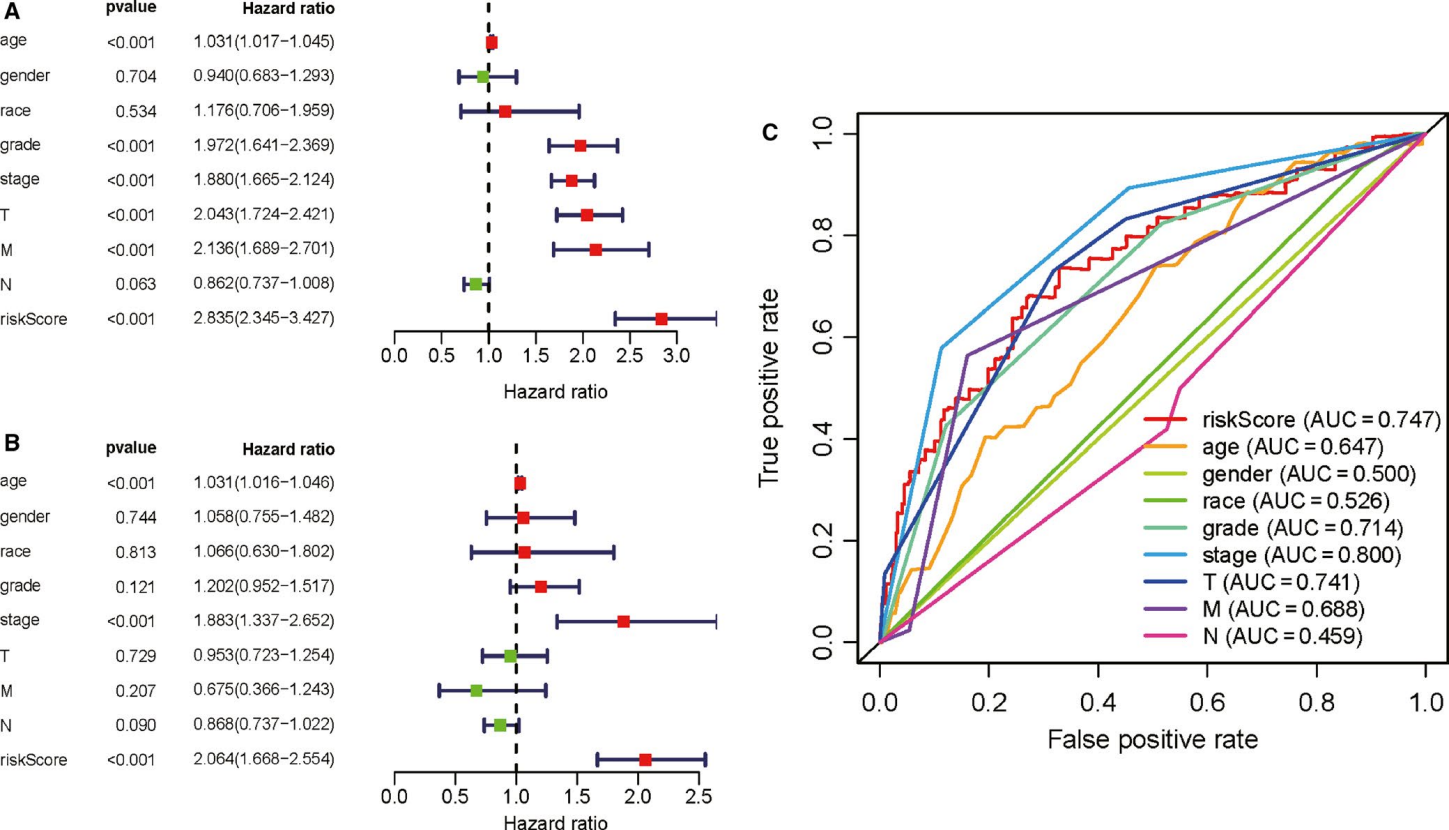
Independent prognostic factor evaluation based on TCGA dataset; A, Univariate cox regression analysis; B, Multivariate cox regression analysis; C, Multiple ROC curves according to risk score, age, gender, race, grade, stage, T, N, M

**Figure 8:**
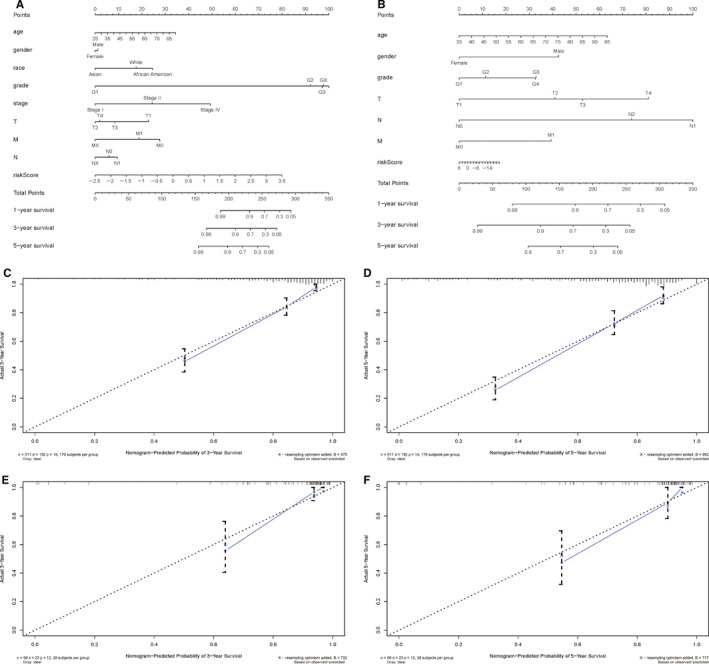
Diagnostic nomograms to clarify the relationship between clinical characters, *riskScore* and prognosis; A, A nomogram for TCGA cohort; B, A nomogram for ArrayExpress cohort; C, the Calibration curve of nomogram‐predicted probability of 3‐year survival based on TCGA cohort; D, the Calibration curve of nomogram‐predicted probability of 5‐year survival based on TCGA cohort; E, the Calibration curve of nomogram‐predicted probability of 3‐year survival based on ArrayExpress cohort; F, the calibration curve of nomogram‐predicted probability of 5‐year survival based on ArrayExpress cohort

To further evaluate whether our model could be used as an independent prognostic factor, we included age, gender, stage, race, grade, T, M, N and *riskScore* as independent variables. By means of univariate and multivariate cox regression analysis, our established PI (*riskScore*) remained significant (both *P* < .001, Figure 7A,B, Table 3). Figure 7C presented the multiple ROC curves according to *riskScore*, age, gender, race, grade, stage, T, N, M. The AUC of the ROC curve made by *riskScore* and stage was among the largest two (0.747 and 0.800 respectively). We next made a prognostic nomogram to quantify the relationship between clinical traits and survival in TCGA cohort and in external cohort, respectively, and its evaluation (Figure 8; Table 2).

**Table 3:** Univariate and multivariate analyses of OS for kidney renal clear cell carcinoma patients based on TCGA

Characteristics	Univariate Cox				Multivariate Cox			
	HR	HR.95L	HR.95H	*P*‐value	HR	HR.95L	HR.95H	*P*‐value
Age	1.03091297	1.0173015	1.04470657	**7.14E‐06**	1.03111693	1.01631139	1.04613815	**3.29E‐05**
Gender	0.93989482	0.68296808	1.2934752	.70359364	1.05777612	0.75494048	1.48209077	.74412445
Race	1.17571796	0.70579892	1.95850782	.53411201	1.06556943	0.63023642	1.8016068	.81264081
Grade	1.97156097	1.64097145	2.36875092	**4.20E‐13**	1.20198028	0.95240081	1.51696279	.12131815
STAGE	1.88044574	1.66452924	2.12437012	**3.38E‐24**	1.88332306	1.33728269	2.65232308	**.00029047**
T	2.04314639	1.7242097	2.42107858	**1.57E‐16**	0.95256084	0.72346258	1.25420746	.7291504
M	2.1356788	1.68860772	2.70111518	**2.42E‐10**	0.67454412	0.36606895	1.24296195	.20676067
N	0.86208791	0.73740524	1.00785231	.06262676	0.86765667	0.73651849	1.02214418	.08951048
*riskScore*	2.83466442	2.34467305	3.42705452	**5.28E‐27**	2.06352682	1.66755103	2.55353082	**2.67E‐11**

Notes

Bold fonts represents that *P* value is <.05.

In order to further evaluate the relationship between 11 prognostic ARGs, *riskScore*, and clinical characteristics, we further made the independent *t* tests. Table 4 detailed the *riskScore* is significantly related to stage, T, M, and grade (*P* < .05). Besides, the correlation between 11 prognosis‐related ARGs and clinical features such as age, gender, stage, race, grade, T, M, N was also analyzed. Bold fonts represented that *P* value was <.05.

**Table 4:** Correlation analysis between 11 prognostic ARGs, *riskScore* and clinical characteristics

ID	Ag e		Gender		Race		Grade		Stage		T		M		N	
	Coef	*P*‐value	Coef	*P*‐value	Coef	*P*‐value	Coef	*P*‐value	Coef	*P*‐value	Coef	*P*‐value	Coef	*P*‐value	Coef	*P*‐value
CASP4	0.429	.668	−1.627	.105	3.819	.148	**−4.145**	**.000**	**−5.615**	**.000**	**−4.951**	**.000**	**−2.845**	**.005**	−0.669	.504
IFNG	−0.081	.936	−0.900	.369	**9.066**	**.011**	**−4.992**	**.000**	**−5.112**	**.000**	**−4.495**	**.000**	**−2.832**	**.005**	0.548	.584
BAG1	−0.451	.652	1.487	.138	**29.483**	**.000**	**5.117**	**.000**	**6.740**	**.000**	**6.478**	**.000**	1.478	.141	−1.062	.289
BNIP3	−0.744	.458	**2.353**	**.019**	5.198	.074	**3.224**	**.001**	**1.967**	**.050**	**2.223**	**.027**	0.217	.828	−0.660	.510
ERBB2	0.532	.595	**2.390**	**.017**	**17.158**	**.000**	**4.826**	**.000**	**5.770**	**.000**	**5.870**	**.000**	1.040	.300	−1.176	.240
RGS19	−0.359	.720	−1.122	.262	0.500	.779	**−5.965**	**.000**	**−5.249**	**.000**	**−4.434**	**.000**	**−2.717**	**.007**	−0.579	.563
BID	−1.152	.250	−1.605	.110	0.327	.849	**−5.110**	**.000**	**−6.542**	**.000**	**−5.611**	**.000**	**−4.407**	**.000**	−0.627	.531
EIF4EBP1	**−2.547**	**.011**	−0.279	.781	3.076	.215	**−5.309**	**.000**	**−6.417**	**.000**	**−5.680**	**.000**	**−4.810**	**.000**	−1.745	.082
CX3CL1	0.706	.481	**3.987**	**.000**	5.456	.065	**4.753**	**.000**	**4.006**	**.000**	**4.420**	**.000**	0.304	.762	−1.011	.312
PRKCQ	0.786	.432	**−2.983**	**.003**	2.306	.316	1.829	.068	**2.197**	**.029**	**2.382**	**.018**	0.239	.812	−1.254	.210
ATG16L2	−0.842	.400	**2.084**	**.038**	**9.989**	**.007**	0.413	.680	−0.877	.381	−0.953	.341	**−2.596**	**.010**	−0.168	.867
*riskScore*	−1.540	.125	−0.501	.617	0.226	.893	**−4.995**	**.000**	**−4.537**	**.000**	**−4.158**	**.000**	**−2.747**	**.007**	−0.293	.770

Notes

Bold fonts represents that *P* value is <.05.

Abbreviations: ARGs, autophagy‐related genes; Coef, correlation coefficient.

### 3.8 Identification of relevant small molecule drugs

cMap database was utilized to screen out candidate small molecule drugs related to ARGs of KIRC. Based on differently expressed ARGs of KIRC, the most significantly related small molecule drugs were identified. *P* < .05, |mean| > 0.5 and n ≥ 4 were set as the threshold. As detailed in Table 5, 10 small molecule drugs were negatively correlated with KIRC containing emetine, cephaeline, co‐dergocrine mesilate, tobramycin, fluvastatin, piribedil, pivampicillin, saquinavir, methylprednisolone, and ifenprodil, indicating the potential to repress this disease. Four small molecule drugs were positively correlated with KIRC containing thioproperazine, copper sulfate, carbachol, and bambuterol, indicating the potential to promote this disease.

**Table 5:** Results of connectivity map (cMap) analysis

Rank	cMap name	Mean	n	Enrichment	*P*	Specificity	Percent on‐null
1	Emetine	−0.654	4	−0.788	.0041	0.0824	100
2	Cephaeline	−0.628	5	−0.78	.0009	0.1145	100
3	Co‐dergocrine mesilate	−0.569	4	−0.762	.00656	0.0226	100
4	Tobramycin	−0.549	4	−0.813	.00229	0	100
5	Fluvastatin	−0.549	4	−0.788	.00408	0	100
6	Piribedil	−0.545	4	−0.781	.00475	0.01	100
7	Pivampicillin	−0.535	4	−0.767	.00593	0	100
8	Saquinavir	−0.527	4	−0.744	.00851	0.0114	100
9	Methylprednisolone	−0.522	4	−0.733	.01026	0.0223	100
10	Ifenprodil	−0.502	4	−0.717	.01313	0.0402	100
11	Thioproperazine	0.547	5	0.826	.00032	0	100
12	Copper sulfate	0.612	4	0.877	.0003	0.0057	100
13	Carbachol	0.613	4	0.897	.0001	0	100
14	Bambuterol	0.728	4	0.872	.00036	0	100

## 4 DISCUSSION

The concept of transformational medicine was first put forward in < lancet>, emphasizing clinical application as the center, to transform the results of basic scientific research into valuable clinical applications.^14^ Currently, surgical resection remained the main method for the treatment of renal cell carcinoma, however these postoperative patients still had a high possibility of recurrence and their survival status varied differently.^15^, ^16^ Hence, an effective way to predict the prognosis of renal cell carcinoma was of great significance to guide the whole process managing patients with renal cell carcinoma.

At present, TMN staging, UISS risk grading system and SSIGN scoring system provided a certain reference value for evaluating the prognosis of renal cell carcinoma patients [^17^, ^18^, ^19^]. Moreover, some prognostic molecular markers such as p53 and PTEN had also been further explored by researchers,^20^ but their efficiency was not so satisfactory. Due to the development of high‐throughput sequencing, it had become feasible for us to use public database (TCGA, GEO or other databases) data for analyzing the associations between different key genes and the clinical outcomes of KIRC. Not long ago, a study by Wang et al have successfully established an autophagy‐clinical prognostic index in bladder cancer patients.^21^ In this article, we not only constructed a prognosis prediction index for KIRC in both TCGA and ArrayExpress databases, but also explored associations between ARGs and TFs. Moreover, we successfully divided KIRC patients into two clusters based on differentially expressed ARGs. Our study was anticipated to provide new insights of autophagy for future work.

We made full use of the RNAseq data in the TCGA database to find autophagy related genes with high correlation with KIRC survival. Finally, we obtained 45 differentially expressed ARGs, and analyzed their functions including GO analysis and KEEG pathways. Preliminary analysis showed that the expression of these ARGs is mostly up‐regulated in some of the most important pathways, which provided us with a reference that autophagy might play a role in promoting tumor development. However, the expressions of some ARGs were down‐regulated, which might be related to the complex mechanism of autophagy in tumors.^22^ Interestingly, we also performed a consensus clustering analysis of existing renal cancer patients based on these ARGs, and KIRC patients were successfully divided into two clusters with significant differences in overall survival, indicating that ARGs might play an important role in the prognosis of patients with KIRC. By means of cMap database, 10 small molecule drugs were negatively correlated with KIRC containing emetine, cephaeline, co‐dergocrine mesilate, tobramycin, fluvastatin, piribedil, pivampicillin, saquinavir, methylprednisolone, and ifenprodil, indicating the potential to repress this disease. Four small molecule drugs were positively correlated with KIRC containing thioproperazine, copper sulfate, carbachol, and bambuterol, indicating the potential to promote this disease.

By means of univariate COX regression analysis, multivariate COX regression analysis and Lasso regression analysis, we ultimately obtained 11 key ARGs (CASP4, IFNG, BAG1, BNIP3, ERBB2, RGS19, BID, EIF4EBP1, CX3CL1, PRKCQ, and ATG16L2). Most of these ARGs had been reported and were consistent with the role in our study. Therein, BNIP3 was an interacting protein of BCL2, which was considered to be a surface receptor of mitochondria, regulating cell death and promoting survival in some diseases.^23^, ^24^ BID played a similar role by activating BAX/BAK ^25^ and ERBB2, also known as HER2, was a member of the human epidermal growth factor receptor family, promoting cell proliferation, survival, and playing an important role in the occurrence and development of tumor. Clinically, ERBB2 mutation had become an important target for cancer therapy. CX3CL1 was the ligand of chemokine CX3CR1, which could promote tumor infiltrating cells into tumor microenvironment and play the role of immunotherapy.^26^ As for IFNG, it was believed that IFNG could affect the blocking of immune checkpoints.^27^ Eukaryotic initiation factor 4e binding protein (EIF4EBP1), as an important gene regulating autophagy, had been found to be highly expressed in many cancers with poor prognosis of tumor.^28^ PRKCQ was an important kinase in the activation of T cells. Its phosphorylation induced the activation of Fra‐1 and played an important role in tumor recurrence and invasion.^29^, ^30^ CASP4, as a kind of human apoptotic protease, was considered to be related to inflammation, immune activity and apoptosis.^31^, ^32^ It has also been found to be related to the poor prognosis of esophageal cancer, colorectal cancer and breast cancer.^33^, ^34^, ^35^ Contrary with the reported results, the HR value of BAG1 was <1 suggesting that it was associated with a better prognosis,.^36^, ^37^ ATG16L2 had been found to be associated with positive prognosis.^38^, ^39^, ^40^ However, the results of our multivariate COX regression analysis suggested that ATG16L2 played an opposite role in KIRC patients. This also brought us new thinking about the roles of BAG1 and ATG16L2 in kidney cancer.

Further, based on these 11 prognostic ARGs and clinical characteristics in both TCGA and ArrayExpress databases, an individualized KIRC PI (*riskScore*) was established. The strength of this article was that we performed a systematic analysis of the roles of autophagy in KIRC with a robust statistical approach. The KIRC PI was successfully established and carefully evaluated in TCGA cohort and ArrayExpress cohort. Moreover, networks between ARGs and TFs were constructed for future basic research. Last but not least, tumor clustering based on ARGs was effective, indicating that ARGs played a vital role in KIRC. However, the limitations of the present article should not be ignored. On the one hand, we only discussed the relationship between ARGs and the prognosis of KIRC patients, without further clarifying the specific mechanism. On the other hand, the results of our study were only validated in the KIRC patient data in the TCGA database and ArrayExpress database. Retrospective data analysis made our prediction model valuable in the training set. Whether it had real application value or not, required more data support from clinical patients.

## 5 CONCLUSIONS

Taken together, an individualized KIRC PI (*riskScore*) was successfully established in both TCGA and ArrayExpress databases. Based on clinical characteristics and 11 key ARGs (CASP4, IFNG, BAG1, BNIP3, ERBB2, RGS19, BID, EIF4EBP1, CX3CL1, PRKCQ, and ATG16L2), our study realized the transformation of a large number of sequencing data and clinical features to the clinical diagnosis and treatment methods. Besides, networks between TFs and ARGs were also displayed and KIRC patients were successfully divided into two clusters based on differentially expressed ARGs. Last but not least, small molecule drugs related to ARGs were also identified for KIRC. Our findings were anticipated to provide new insights of autophagy for future work.

## CONFLICT OF INTEREST

None declared.

## AUTHORS CONTRIBUTIONS

Y.W: Protocol/project development; R.C: Data collection or management; BY.Z: Data analysis; QW.X, CJ.J: Manuscript writing/editing.

## ETHICS APPROVAL

Not applicable.

## CONSENT FOR PUBLICATION

Not applicable.

## Supporting information

Fig S1

Fig S2

Supplementary Material

Supplementary Material

Supplementary Material

## Data Availability

All the data used to support the findings of this study are included within the article. Please contact author for data requests.

## References

[1] Kroemer G , Marino G , Levine B . Autophagy and the integrated stress response. Mol Cell. 2010;40:280‐293. 20965422 10.1016/j.molcel.2010.09.023 PMC3127250

[2] Dutta S , Warshall C , Bandyopadhyay C , Dutta D , Chandran B . Interactions between exosomes from breast cancer cells and primary mammary epithelial cells leads to generation of reactive oxygen species which induce DNA damage response, stabilization of p53 and autophagy in epithelial cells. PLoS ONE. 2014;9:e97580. 24831807 10.1371/journal.pone.0097580 PMC4022578

[3] Eisenberg‐Lerner A , Kimchi A . The paradox of autophagy and its implication in cancer etiology and therapy. Apoptosis. 2009;14:376‐391. 19172397 10.1007/s10495-008-0307-5

[4] Nassour J , Radford R , Correia A , et al. Autophagic cell death restricts chromosomal instability during replicative crisis. Nature. 2019;565:659‐663. 30675059 10.1038/s41586-019-0885-0 PMC6557118

[5] White E . The role for autophagy in cancer. J Clin Invest. 2015;125:42‐46. 25654549 10.1172/JCI73941 PMC4382247

[6] Tan Q , Wang M , Yu M , et al. Role of autophagy as a survival mechanism for hypoxic cells in tumors. Neoplasia. 2016;18:347‐355. 27292024 10.1016/j.neo.2016.04.003 PMC4909700

[7] Das CK , Linder B , Bonn F , et al. BAG3 overexpression and cytoprotective autophagy mediate apoptosis resistance in chemoresistant breast cancer cells. Neoplasia. 2018;20:263‐279. 29462756 10.1016/j.neo.2018.01.001 PMC5852393

[8] Das CK , Parekh A , Parida PK , Bhutia SK , Mandal M . Lactate dehydrogenase A regulates autophagy and tamoxifen resistance in breast cancer. Mol Cell Res. 1866;2019:1004‐1018. 10.1016/j.bbamcr.2019.03.004 30878502

[9] Bray F , Ferlay J , Soerjomataram I , Siegel RL , Torre LA , Jemal A . Global cancer statistics 2018: GLOBOCAN estimates of incidence and mortality worldwide for 36 cancers in 185 countries. CA Cancer J Clin. 2018;68:394‐424. 30207593 10.3322/caac.21492

[10] Zheng T , Yang CG . Targeting SPOP with small molecules provides a novel strategy for kidney cancer therapy. Sci China Life Sci. 2017;60:91‐93. 27888385 10.1007/s11427-016-0297-2

[11] De Meerleer G , Khoo V , Escudier B , et al. Radiotherapy for renal‐cell carcinoma. Lancet Oncol. 2014;15:e170‐e177. 10.1016/S1470-2045(13)70569-2 24694640

[12] Jin L , Xu X , Ye B , Pan M , Shi Z , Hu Y . Elevated serum interleukin‐35 levels correlate with poor prognosis in patients with clear cell renal cell carcinoma. Int J Clin Exp Med. 2015;8:18861‐18866. 26770508 PMC4694408

[13] Lamb J , Crawford ED , Peck D , et al. The connectivity map: using gene‐expression signatures to connect small molecules, genes, and disease. Science. 2006;313:1929‐1935. 17008526 10.1126/science.1132939

[14] Meeks JJ , Goldkorn A , Aparicio AM , McConkey DJ . Development of a translational medicine protocol for an NCTN genitourinary clinical trial: critical steps, common pitfalls and a basic guide to translational clinical research. Urol Oncol. 2019;37:313‐317. 30115512 10.1016/j.urolonc.2018.06.008 PMC6886001

[15] Janzen NK , Kim HL , Figlin RA , Belldegrun AS . Surveillance after radical or partial nephrectomy for localized renal cell carcinoma and management of recurrent disease. Urol Clin North Am. 2003;30:843‐852. 14680319 10.1016/s0094-0143(03)00056-9

[16] Leibovich BC , Blute ML , Cheville JC , et al. Prediction of progression after radical nephrectomy for patients with clear cell renal cell carcinoma: a stratification tool for prospective clinical trials. Cancer. 2003;97:1663‐1671. 12655523 10.1002/cncr.11234

[17] Klatte T , Rossi SH , Stewart GD . Prognostic factors and prognostic models for renal cell carcinoma: a literature review. World J Urol. 2018;36:1943‐1952. 29713755 10.1007/s00345-018-2309-4

[18] Zisman A , Pantuck AJ , Wieder J , et al. Risk group assessment and clinical outcome algorithm to predict the natural history of patients with surgically resected renal cell carcinoma. J Clin Oncol. 2002;20:4559‐4566. 12454113 10.1200/JCO.2002.05.111

[19] Borghesi M , Brunocilla E , Schiavina R , Martorana G . Positive surgical margins after nephron‐sparing surgery for renal cell carcinoma: incidence, clinical impact, and management. Clin Genit Cancer. 2013;11:5‐9. 10.1016/j.clgc.2012.09.010 23083800

[20] Ingels A , Hew M , Algaba F , et al. Vimentin over‐expression and carbonic anhydrase IX under‐expression are independent predictors of recurrence, specific and overall survival in non‐metastatic clear‐cell renal carcinoma: a validation study. World J Urol. 2017;35:81‐87. 27207480 10.1007/s00345-016-1854-y

[21] Wang SS , Chen G , Li SH , et al. Identification and validation of an individualized autophagy‐clinical prognostic index in bladder cancer patients. Onco Targets Ther. 2019;12:3695‐3712. 31190871 10.2147/OTT.S197676 PMC6526186

[22] Li X , Zhou Y , Li Y , et al. Autophagy: a novel mechanism of chemoresistance in cancers. Biomed Pharm. 2019;119:109415. 10.1016/j.biopha.2019.109415 31514065

[23] Li R , Xin T , Li D , Wang C , Zhu H , Zhou H . Therapeutic effect of Sirtuin 3 on ameliorating nonalcoholic fatty liver disease: the role of the ERK‐CREB pathway and Bnip3‐mediated mitophagy. Redox Biol. 2018;18:229‐243. 30056271 10.1016/j.redox.2018.07.011 PMC6079484

[24] Zhang T , Xue L , Li L , et al. BNIP3 protein suppresses PINK1 kinase proteolytic cleavage to promote mitophagy. J Biol Chem. 2016;291:21616‐21629. 27528605 10.1074/jbc.M116.733410 PMC5076832

[25] Lovell JF , Billen LP , Bindner S , et al. Membrane binding by tBid initiates an ordered series of events culminating in membrane permeabilization by Bax. Cell. 2008;135:1074‐1084. 19062087 10.1016/j.cell.2008.11.010

[26] Nagarsheth N , Wicha MS , Zou W . Chemokines in the cancer microenvironment and their relevance in cancer immunotherapy. Nat Rev Immunol. 2017;17:559‐572. 28555670 10.1038/nri.2017.49 PMC5731833

[27] Benci JL , Johnson LR , Choa R , et al. Opposing functions of interferon coordinate adaptive and innate immune responses to cancer immune checkpoint blockade. Cell. 2019;178:933‐948.e914. 31398344 10.1016/j.cell.2019.07.019 PMC6830508

[28] Rutkovsky AC , Yeh ES , Guest ST , et al. Eukaryotic initiation factor 4E‐binding protein as an oncogene in breast cancer. BMC Cancer. 2019;19:491. 31122207 10.1186/s12885-019-5667-4 PMC6533768

[29] Belguise K , Cherradi S , Sarr A , et al. PKCtheta‐induced phosphorylations control the ability of Fra‐1 to stimulate gene expression and cancer cell migration. Cancer Lett. 2017;385:97‐107. 27816489 10.1016/j.canlet.2016.10.038

[30] Belguise K , Milord S , Galtier F , Moquet‐Torcy G , Piechaczyk M , Chalbos D . The PKCtheta pathway participates in the aberrant accumulation of Fra‐1 protein in invasive ER‐negative breast cancer cells. Oncogene. 2012;31:4889‐4897. 22286759 10.1038/onc.2011.659 PMC3624663

[31] Jorgensen I , Miao EA . Pyroptotic cell death defends against intracellular pathogens. Immunol Rev. 2015;265:130‐142. 25879289 10.1111/imr.12287 PMC4400865

[32] McIlwain DR , Berger T , Mak TW . Caspase functions in cell death and disease. Cold Spring Harbor Perspect Biol. 2013;5:a008656. 10.1101/cshperspect.a008656 PMC3683896 23545416

[33] Zhu X , Li S . TET2 inhibits tumorigenesis of breast cancer cells by regulating caspase‐4. Sci Rep. 2018;8:16167. 30385776 10.1038/s41598-018-34462-z PMC6212556

[34] Soung YH , Jeong EG , Ahn CH , et al. Mutational analysis of caspase 1, 4, and 5 genes in common human cancers. Hum Pathol. 2008;39:895‐900. 18430458 10.1016/j.humpath.2007.10.015

[35] Shibamoto M , Hirata H , Eguchi H , et al. The loss of CASP4 expression is associated with poor prognosis in esophageal squamous cell carcinoma. Oncol Lett. 2017;13:1761‐1766. 28454321 10.3892/ol.2017.5646 PMC5403698

[36] Clemo NK , Collard TJ , Southern SL , et al. BAG‐1 is up‐regulated in colorectal tumour progression and promotes colorectal tumour cell survival through increased NF‐kappaB activity. Carcinogenesis. 2008;29:849‐857. 18204076 10.1093/carcin/bgn004

[37] Gennaro VJ , Wedegaertner H , McMahon SB . Interaction between the BAG1S isoform and HSP70 mediates the stability of anti‐apoptotic proteins and the survival of osteosarcoma cells expressing oncogenic MYC. BMC Cancer. 2019;19:258. 30902071 10.1186/s12885-019-5454-2 PMC6429775

[38] Mo S , Dai W , Xiang W , et al. Prognostic and predictive value of an autophagy‐related signature for early relapse in stages I‐III colon cancer. Carcinogenesis. 2019;40:861‐870. 30933267 10.1093/carcin/bgz031

[39] Yuan J , Zhang N , Yin L , et al. Clinical implications of the autophagy core gene variations in advanced lung adenocarcinoma treated with Gefitinib. Sci Rep. 2017;7:17814. 29259263 10.1038/s41598-017-18165-5 PMC5736620

[40] Wen J , Liu H , Wang L , et al. Potentially functional variants of ATG16L2 predict radiation pneumonitis and outcomes in patients with non‐small cell lung cancer after definitive radiotherapy. J Thorac Oncol. 2018;13:660‐675. 29454863 10.1016/j.jtho.2018.01.028 PMC6124306

