# Supplementary figures and images for: Identification of small molecule drugs and development of a novel autophagy‐related prognostic signature for kidney renal clear cell carcinoma
Source: Cancer Med. 2020 Aug 11;9(19):7034–51. doi: 10.1002/cam4.3367 (PMC7541166; doi:10.1002/cam4.3367)

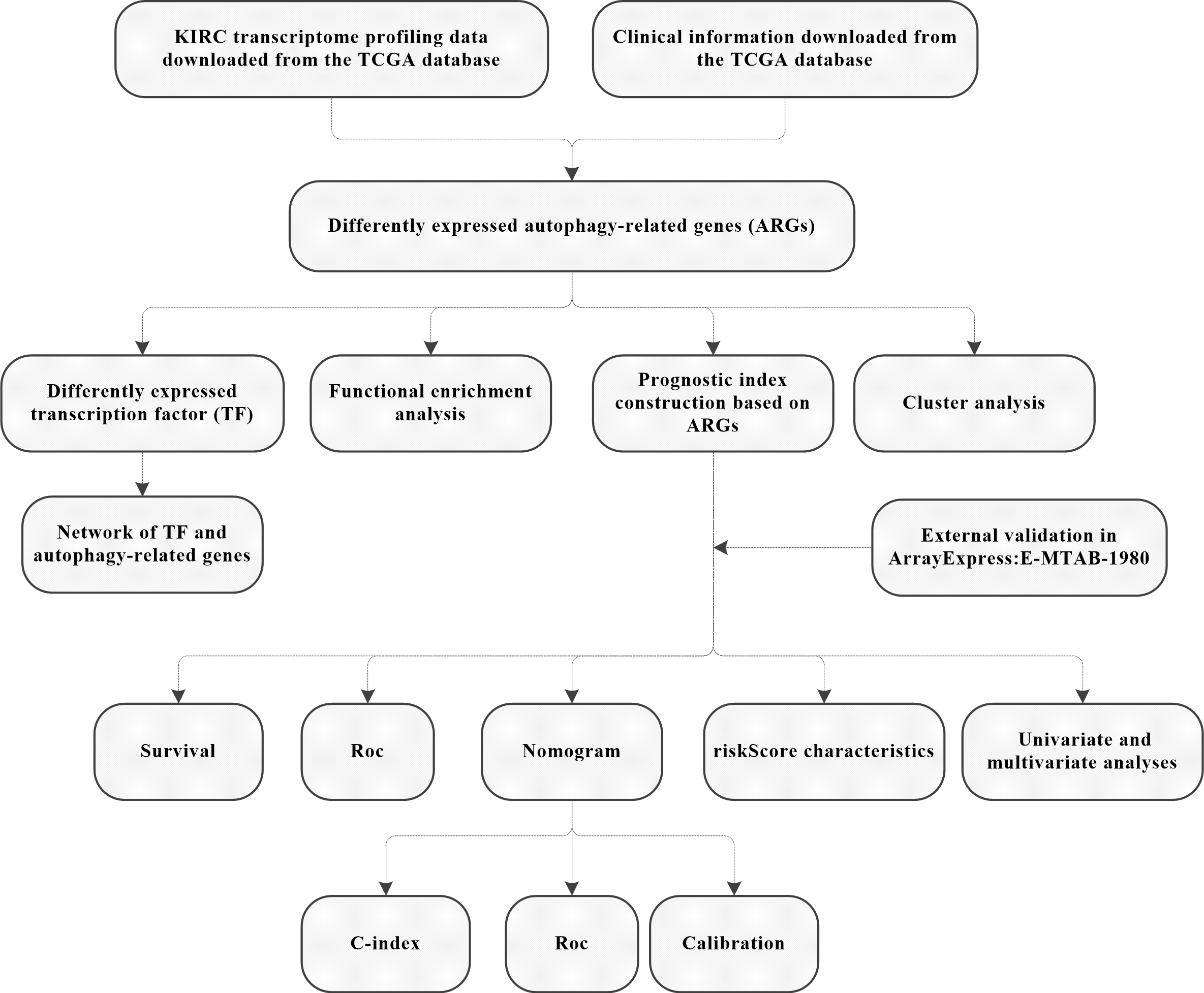

Supplement: Supplementary file 1 — Fig S1 [file CAM4-9-7034-s001.tif]

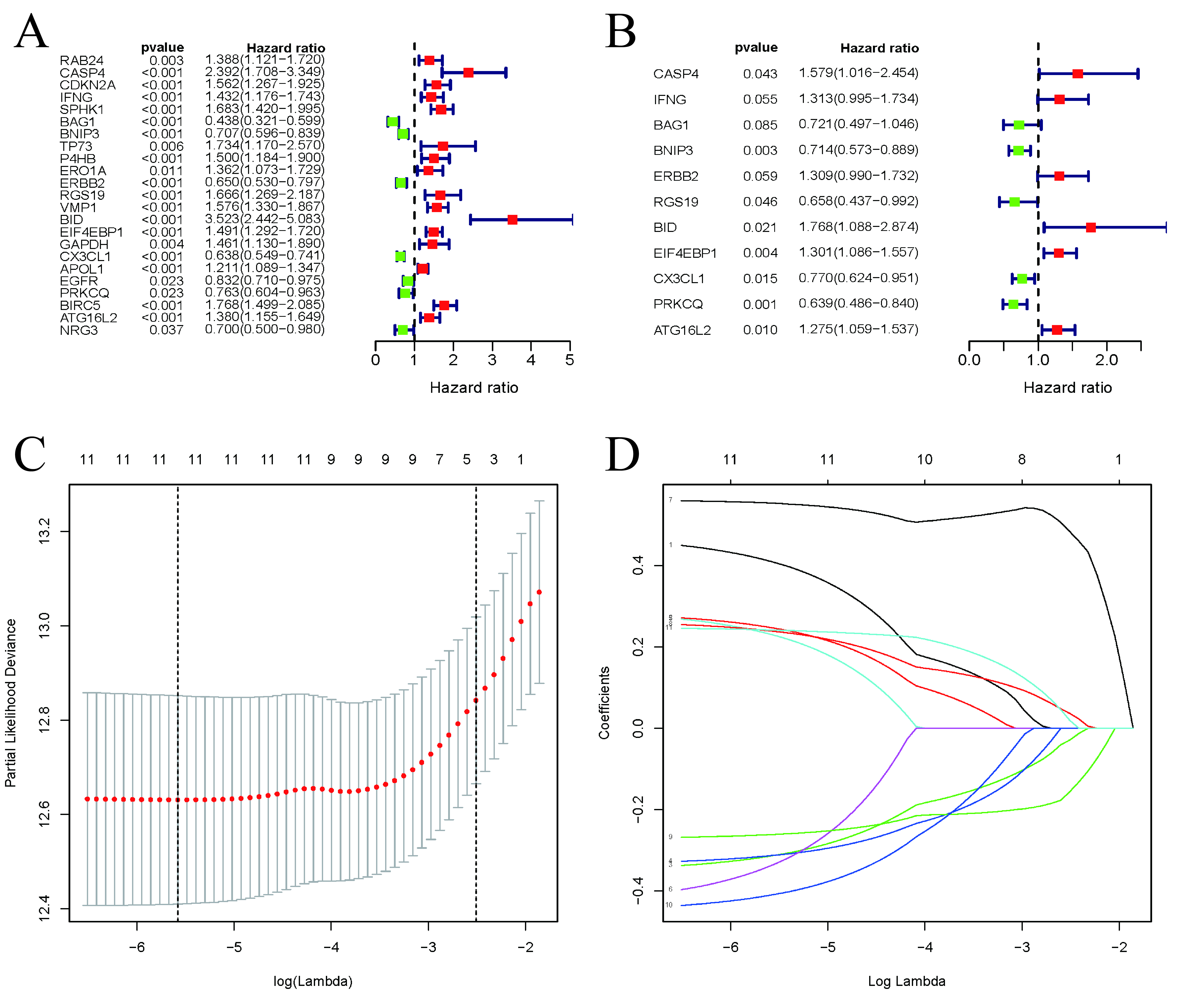

Supplement: Supplementary file 2 — Fig S2 [file CAM4-9-7034-s002.tif]
